# Real-World Incidence of Anaplastic Lymphoma Kinase Alterations in Hispanics with Non–Small Cell Lung Cancer at a Large Academic Institution in Los Angeles

**DOI:** 10.1158/2767-9764.CRC-24-0504

**Published:** 2025-02-10

**Authors:** Darin Poei, Sana Ali, Jacob S. Thomas, Jorge J. Nieva, Robert C. Hsu

**Affiliations:** 1 Department of Medicine, University of Southern California, Los Angeles, California.; 2 Division of Medical Oncology, Department of Medicine, University of Southern California Norris Comprehensive Cancer Center, Los Angeles, California.

## Abstract

Race and ethnicity affect the distribution of molecular alterations seen in patients with non–small cell lung cancer (NSCLC). Although anaplastic lymphoma kinase (ALK) alterations are known to occur in 4% to 5% of the population, data specific to the Hispanic population remain limited. This study describes the real-world incidence of ALK alterations in Hispanic patients with NSCLC treated at a large academic institution in Los Angeles, California, United States, to further elucidate the underlying factors that shape differences in mutational profiles. A total of 607 patients with NSCLC treated at Los Angeles General Medical Center (*n* = 172) and the University of Southern California Norris Comprehensive Cancer Center (*n* = 435) who received comprehensive genomic profiling were evaluated from January 2015 to June 2023. The Fisher exact test and multivariate logistic regression were incorporated for statistical analysis. Hispanic patients exhibited a higher incidence of ALK alterations (12.76%, *n* = 18/141) than non-Hispanic patients (5.36%, *n* = 23/466; *P* = 0.0046). Multivariate logistic regression showed that Hispanic ethnicity (HR, 2.393; 95% confidence interval, 1.115–5.092) and age at diagnosis (HR, 0.9325; 95% confidence interval, 0.9081–0.9558) were significant variables in ALK alteration incidence, and 55.81% (*n* = 24) of ALK alterations were initially found via comprehensive genomic profiling. This study highlights a significantly higher incidence of ALK alterations among Hispanic patients with NSCLC, emphasizing the impact of race and ethnicity on molecular alterations. Future research is required to evaluate this trend in a larger, more diverse cohort and investigate the roles of environmental factors and tumor microenvironments in influencing ALK alteration prevalence.

**Significance:**

This study identified a higher incidence of ALK alterations in Hispanic patients with NSCLC (12.76%) compared with that in non-Hispanic patients (5.36%) treated at a large academic center in Los Angeles, highlighting the impact of race on molecular alteration profiles and emphasizing the need to increase access to molecular analyses for this population. The variability in mutational alterations may be influenced by biological and environmental factors.

## Introduction

As per the American Cancer Society (RRID: SCR_005756) and Surveillance Epidemiology and End Results (SEER; RRID: SCR_006902) program, lung cancer remains the third most common type of cancer in the United States, accounting for 11.7% of all new cancer diagnoses, with non–small cell lung cancer (NSCLC) comprising 80% to 85% of cases. It is estimated that 234,580 patients will be diagnosed with lung cancer and 125,070 patients will die of the disease in 2024 (as per SEER; RRID: SCR_006902). According to the U.S. Census Bureau (RRID: SCR_011587), the Hispanic or Latino population is the largest ethnic or racial minority in the United States, comprising 19.1% of the total population, and this number is projected to increase to 31% of the total U.S. population by 2060. With regard to lung cancer, Hispanic males and females account for lower rates of new lung cancer cases (31.0 and 22.6 per 100,000, respectively) when compared with non-Hispanic White males and females (60.0 and 52.2 per 100,000, respectively; as per SEER; RRID: SCR_006902). Between 2018 and 2022, the death rates from lung cancer in Hispanic males and females were 19.4 and 11.1 deaths per 100,000, respectively (as per SEER; RRID: SCR_006902). Despite the clinical significance of lung cancer in Hispanic or Latino patients, significant disparities in genomic mutational analyses and clinical trial enrollment persist (1, 2). Since the discovery of actionable mutations, including the rearrangements of anaplastic lymphoma kinase (ALK) and echinoderm microtubule–associated protein–like 4 (EML4) genes, targeted therapies have dramatically improved survival outcomes in patients with NSCLC. In 2007, the EML4–ALK rearrangement was first identified when 7% of a small cohort of Japanese patients with NSCLC was found to harbor the EML4 rearrangement, which fused to ALK, leading to the creation of the oncogene EML4–ALK (3). The ALK gene rearrangement is found in approximately 5% of patients with NSCLC (3). This gene rearrangement creates an activated ALK tyrosine kinase that leads to cell proliferation and survival, which serves as a driver for tumor formation (4). Patients who develop this alteration tend to be younger and light or never smokers and are most commonly diagnosed with adenocarcinoma (5).

Given the existence of various break points within the EML4 gene, multiple variants of the EML4–ALK rearrangement have been described in literature (6, 7). It has been shown that variant 1 (45%–54.5%) is the most common subtype, followed by variant 2 (34.0%–34.5%) and then variant 3 (10.0%–14.5%; refs. 7, 8). As it pertains to survival, it has been observed that patients with variant 3 had worse survival outcomes when compared with those with variants 1 and 2 (9–11). In a study of 116 patients with ALK-rearranged NSCLC from five Latin American cancer centers, variant 3a/b was the most predominant subtype (36%), followed by variant 1 (12). The median tumor mutation burden in this cohort was 1 mutation/mb (range, 1–4; ref. 12). It is noteworthy that these studies investigating EML4–ALK variants have largely been conducted outside of the United States, highlighting the need for further research.

The ALK rearrangement serves an important role as a therapeutic target in the treatment of patients with NSCLC. Typically, these patients are given an oral medication, which are tyrosine kinase inhibitors targeting the ALK gene rearrangement. These ALK tyrosine kinase inhibitors have shown significant benefits in overall and progression-free survival for patients with ALK-positive (ALK^+^) NSCLC when compared with chemotherapy (13). In the past decade, several generations of ALK inhibitors have been approved for standard-of-care management of the ALK rearrangement (14–16). As such, early and reliable detection of ALK rearrangements in patients with NSCLC is key for diagnosis and management. There are multiple ways of detecting ALK rearrangements, which include FISH, IHC, and next-generation sequencing (NGS; ref. 17). Molecular testing often involves multiple assays, although multigene panels can identify the most common alterations in a single assay.

Although the current literature published on ALK-rearranged NSCLC is expansive, little information is published on its incidence in the Hispanic population despite the fact that Hispanics comprise 18.9% of the U.S. population and account for 49.1% of those in Los Angeles County (as per U.S. Census Bureau; RRID: SCR_011587). One prior study of patients with NSCLC in Latin America demonstrated significant variability in the incidence of ALK rearrangements between countries, regions, and continents, suggesting a key role for race and ethnicity (18). Prior studies have observed a frequency of ALK rearrangements ranging from 1.5% to 10.5% in the general Hispanic/Latino population (19–22). A previous study from our group found a trend toward higher prevalence of ALK rearrangements among Hispanic patients when compared with Asian patients (23). Despite these studies, there remains a considerable lack of data about the mutational subtypes seen in Hispanic patients with NSCLC, specifically ALK alterations. This study aims to describe the real-world incidence of ALK alterations in Hispanic patients with NSCLC at a large academic institution in Los Angeles. Additionally, the study will discuss potential factors that may contribute to the variability of molecular alterations observed between different races and ethnicities.

## Materials and Methods

### Study population and eligibility

This study consisted of patients with pathologically confirmed NSCLC treated at the Los Angeles General Medical Center (LAG), a safety-net hospital for the County of Los Angeles, and the University of Southern California Norris Comprehensive Cancer Center (Norris), an NCI-designated comprehensive cancer center. The inclusion criteria were that patients must have pathologically confirmed NSCLC of either adenocarcinoma, squamous, or large cell histology and that patients must have been treated at the LAG or Norris. The exclusion criteria were that patients with small cell lung cancer were not eligible. Data were collected from January 1, 2015, to June 30, 2023. The study was approved by the University of Southern California Institutional Review Board. Data were obtained from patients’ electronic medical records via retrospective chart review and stored into a password-protected data file. Data were reviewed in a secure file, and only Institutional Review Board–approved study personnel accessed the data. Informed consent was waived given the retrospective nature of the study.

### Molecular testing

Patients with advanced NSCLC were included if they received molecular testing to identify the presence of molecular alterations to guide treatment decisions. Patients received either a la carte testing or comprehensive genomic profiling (CGP) via a biopsy of the tissue from the tumor (tissue biopsy) or a blood test (liquid biopsy). A la carte testing was defined as the testing of single genes (typically 3–4 genes consisting of *EGFR* using Sanger sequencing or NGS, ALK using FISH or PCR, *ROS1* using FISH, and *BRAF* using NGS) primarily through Quest Diagnostics (Quest Diagnostics, *EGFR* testing: https://testdirectory.questdiagnostics.com/test/test-detail/16460/epidermal-growth-factor-receptor-egfr-mutation-analysis?cc=MASTER; ALK testing: https://testdirectory.questdiagnostics.com/test/test-detail/91028/fish-alk-2p23-rearrangement-lung-cancer-nsclc?cc=MASTER; *ROS1* testing: https://testdirectory.questdiagnostics.com/test/test-detail/91836/lung-cancer-nsclc-ros1-6q22-rearrangement-fish?cc=MASTER; and BRAF testing: https://testdirectory.questdiagnostics.com/test/test-detail/16767/braf-mutation-analysis?cc=MASTER). Tissue biopsies obtained for CGP were sent to Caris Life Sciences or Tempus and liquid biopsies were sent to Guardant Health or Tempus for analysis. Caris Life Sciences utilizes tissue-based whole-exome and whole-transcriptome sequencing analyses via NGS to identify all of the actionable mutations, IHC to help identify PD-L1 status, pyrosequencing, and chromogenic in situ hybridization (CISH) on tissue. Caris Molecular Intelligence (MI) profile comprehensive testing tests for more than 23,000+ genes and includes artificial intelligence (AI) predictive algorithms for cancers of unknown primary origin and metastatic colorectal cancer. The reports come with results that include therapy association and biomarker levels (level 1: biomarker test noted in FDA indication, level 2: endorsed by clinical guidelines, and level 3: evidence exists in patient’s tumor type), therapies with potential benefit/lack of benefit, cancer-type–relevant biomarkers, and clinical trial information (Caris Life Sciences, https://www.ncbi.nlm.nih.gov/gtr/tests/560898/). Tempus xT testing uses a 648-gene DNA sequencing panel that reports clinically relevant alterations, involves IHC testing for immunotherapy biomarkers, and includes RNA sequencing (Tempus, https://www.ncbi.nlm.nih.gov/gtr/tests/558436/). Testing at Guardant Health consisted of a Guardant 360 CDx panel which is an NGS test that uses circulating cell-free DNA from plasma of peripheral whole blood collected in Streck Cell-Free DNA Blood Collection Tubes to detect single-nucleotide variants, insertions and deletions in 55 genes, copy-number amplifications in two genes, and fusions in four genes, including ALK fusions (Guardant Health, https://www.ncbi.nlm.nih.gov/gtr/tests/527948/). Tempus xF liquid biopsy assay is a noninvasive 105-gene, hybrid-capture, NGS assay that is focused on oncogenic and resistance mutations in cell-free DNA that detects single-nucleotide variants, copy-number amplifications, and chromosomal rearrangements. The DNA sequencing depth was an average of 20,000× with raw reads and 5,000× with unique reads (Tempus, https://www.ncbi.nlm.nih.gov/gtr/tests/569040/).

### Data collection

Data obtained included both demographic data (age, sex, race/ethnicity, and smoking history) and clinical characteristics at diagnosis (stage, histology, brain metastasis, and presence of targetable mutations). Race/ethnicity and smoking history were self-reported by patients. Patients were classified as Asian, Hispanic, non-Hispanic White, non-Hispanic Black, or other/unknown. The presence of targetable mutations was determined by both a la carte testing as well as CGP. To help contextualize data from our institution, we used data from the U.S. Census Bureau (RRID: SCR_011587) in our literature search to evaluate the percentage of Hispanics in Los Angeles County. We also used publicly available data from the California Cancer Registry using CAL*Explorer (RRID: SCR_026228). We also obtained general background information on patients with lung cancer from the American Cancer Society (RRID: SCR_005756) and SEER (RRID: SCR_006902).

### Statistical analysis

The Fisher exact test was performed to compare the differences in the prevalence of targetable mutations between the different races/ethnicities. Multivariate logistic regression was performed to evaluate the role of Hispanic ethnicity when controlling for the site of treatment, age at initial diagnosis, sex, and smoking history. Statistical analysis was performed using GraphPad Prism 10 software GraphPad Prism (RRID: SCR_002798). A *P* value of <0.05 was considered statistically significant.

### Data reporting

Race/ethnicity data were self-reported and based on the information listed in the electronic medical record, and there were 39 patients listed as “other” and 7 patients with unknown race/ethnicity data in our study. Smoking history was obtained from chart review and found either in the social history or in the notes. Molecular testing results were obtained via the reports obtained from the companies providing the molecular testing. There were limitations with regards to the clinical history of some patients, particularly those who had received initial care outside of the LAG and Norris, as some of the staging at diagnosis was incomplete, and as a result, the patients were listed as “early stage” if the tumor–node–metastasis staging for nonmetastatic patients was unknown. There were also patients who received molecular testing but were lost to follow-up after.

### Data availability

The data generated in this study are available upon request from the corresponding author.

## Results

This study consisted of 607 patients with NSCLC who received care at the LAG (*n* = 172) and Norris (*n* = 435). Of the 607 patients, 294 (48.43%) were female. The median age at diagnosis of the total sample was 65.7 years, and 23.23% (*n* = 141) identified as Hispanic, 33.61% (*n* = 204) as Asian, 5.77% (*n* = 35) as non-Hispanic Black, 29.98% (*n* = 182) as non-Hispanic White, and 7.41% (*n* = 45) as other/unknown. Among the Hispanic patients (*n* = 141), 46.80% were female and 53.90% were nonsmokers. In the Asian population (*n* = 204), 51.47% were female and 59.80% were nonsmokers (Table 1).

**Table 1:** Summary of demographic features of patients with NSCLC treated at the LAG and Norris

Demographic feature	Hispanic	Asian	Non-Hispanic Black	Non-Hispanic White	Other/unknown	Total
Total population	141 (23.23)	204 (33.61)	35 (5.77)	182 (29.98)	45 (7.41)	607
Median age at diagnosis (years)	62.1	66.1	63.6	68.4	65.6	65.7
Males (%)	75 (53.19)	99 (48.43)	21 (60.00)	93 (51.10)	25 (55.56)	313 (51.57)
Females (%)	66 (46.80)	105 (51.47)	14 (40.00)	89 (48.90)	20 (44.44)	294 (48.43)
Never smokers (%)	76 (53.90)	122 (59.80)	8 (22.86)	55 (30.22)	19 (42.22)	280 (46.13)
Former smokers (%)	48 (34.04)	71 (34.80)	18 (51.43)	103 (56.59)	24 (53.33)	264 (43.49)
Current smokers (%)	17 (12.06)	11 (5.39)	9 (25.71)	24 (13.19)	2 (4.44)	63 (10.38)

For the Hispanic population, 80.85% were found to have adenocarcinoma. Among Hispanic patients, 73.05% were found to have stage IV disease at diagnosis, and 46 patients (32.62%) were found to have brain metastasis at diagnosis (Table 2).

**Table 2:** Prevalence of adenocarcinoma histology, stage at diagnosis, and metastasis to brain by race/ethnicity

	Hispanic (*n* = 141)	Asian (*n* = 204)	Non-Hispanic Black (*n* = 35)	Non-Hispanic White (*n* = 182)	Other/unknown (*n* = 45)	Total (*n* = 607)
Adenocarcinoma (%)	114 (80.85)	163 (79.90)	25 (71.43)	121 (66.48)	31 (68.89)	454 (74.79)
Stage I at diagnosis (%)	11 (7.80)	35 (17.16)	4 (11.43)	28 (15.38)	8 (17.78)	86 (14.17)
Stage II at diagnosis (%)	8 (5.67)	18 (8.82)	5 (14.29)	19 (10.44)	3 (6.67)	53 (8.73)
Stage III at diagnosis (%)	19 (13.48)	35 (17.16)	10 (28.57)	39 (21.43)	12 (26.67)	115 (18.95)
Early stage at diagnosis (%)	—	1 (0.49)	—	1 (0.55)	—	2 (0.33)
Stage IV at diagnosis (%)	103 (73.05)	115 (56.37)	16 (45.71)	95 (52.20)	22 (48.89)	351 (57.83)
Brain metastasis at diagnosis (%)	46 (32.62)	60 (29.41)	11 (31.43)	40 (21.98)	6 (13.33)	163 (26.85)

Among patients with NSCLC, 7.08% (*n* = 43) were found to have an ALK rearrangement on CGP. Among these patients, 12.76% (*n* = 18) self-identified as Hispanic, 4.41% (*n* = 9) as Asian, 0% (*n* = 0) as non-Hispanic Black, 14 (7.69%) as non-Hispanic White, and 4.44% (*n* = 2) as other/unknown. When compared with non-Hispanic patients (5.36%, *n* = 25/466), Hispanic patients were significantly more likely to harbor ALK alterations (12.76%, *n* = 18/141; *P* = 0.0046). The most common histology seen in ALK^+^ NSCLC was adenocarcinoma (86.05%; *n* = 37). Among 18 Hispanic patients with ALK alterations, 50% of patients were female (*n* = 9) and the median age at diagnosis was 52.75 years, whereas in all Hispanic patients, 46.80% (*n* = 66/141) were female and the median age at diagnosis was 62.1 years (Tables 1 and 3). Among Hispanic ALK patients, 61.11% (*n* = 11) were never smokers compared with 65.90% (*n* = 29) in all patients with ALK alterations. Among Hispanic ALK patients, 94.44% (*n* = 17) were diagnosed with stage IV disease compared with 72.34% (*n* = 102) in all Hispanics. Brain metastases were seen in 61.11% (*n* = 11) of Hispanic ALK patients compared with 32.62% (*n* = 46) in all Hispanics (Table 3).

**Table 3:** Prevalence of ALK alterations, median age at diagnosis, prevalence of males and females, never smokers, stage IV at diagnosis, and brain metastasis in the ALK population by race/ethnicity

	Hispanic (*n* = 141)	Asian *(n* = 204)	Non-Hispanic Black (*n* = 35)	Non-Hispanic White (*n* = 182)	Other/unknown (*n* = 45)	Total (*n* = 607)
Number of ALK alterations (%)	18 (12.76)	9 (4.41)	0 (0.00)	14 (7.69)	2 (4.44)	43 (7.08)
Median age at diagnosis of the ALK population	52.75	46.05	—	53.7	51.5	53
Males in the ALK population (%)	9 (50.00)	5 (55.56)	—	6 (42.86)	1 (50.00)	21 (47.72)
Females in the ALK population (%)	9 (50.00)	4 (44.44)	—	8 (57.14)	1 (50.00)	23 (52.27)
Never smokers in the ALK population (%)	11 (61.11)	6 (66.67)	—	9 (64.29)	1 (50.00)	27 (62.79)
Never smoker in the total population (%)	76 (53.90)	122 (59.80)	8 (22.86)	55 (30.22)	19 (42.22)	280 (46.13)
Adenocarcinoma in the ALK population (%)	17 (94.44)	7 (77.78)	—	13 (92.86)	0 (0.00)	37 (86.05)
Adenocarcinoma in the total population (%)	114 (80.85)	163 (79.90)	25 (71.43)	121 (66.48)	31 (68.89)	454 (74.79)
Stage IV at diagnosis in the ALK population (%)	17 (94.44)	6 (66.67)	—	13 (86.67)	2 (100.00)	38 (88.37)
Stage IV at diagnosis in the total population (%)	103 (73.05)	115 (56.37)	16 (45.71)	95 (52.20)	22 (48.89)	351 (57.83)
Brain metastasis in the ALK population (%)	11 (61.11)	2 (22.22)	—	8 (53.33)	2 (100.00)	23 (52.28)
Brain metastasis in the total population (%)	46 (32.62)	60 (29.41)	11 (31.43)	40 (21.98)	6 (13.33)	163 (26.85)

	Hispanic (*n* = 5)	Asian (*n* = 4)	Non-Hispanic Black (*n* = 0)	Non-Hispanic White (*n* = 5)	Other/unknown (*n* = 1)	Total (*n* = 15)
Variant 1	4	2	0	4	0	10
Variant 2	0	0	0	0	1	1
Variant 3	1	2	0	1	0	4

Of this cohort, the specific variant subtypes for 13 patients were documented in electronic medical record (EMR). Ten patients (66.67%) were found to harbor EML4–ALK variant 1, whereas four patients (26.66%) were found to have EML4–ALK variant 3, and one patient (6.67%) had EML4–ALK variant 2. Eighteen of the patients were found to have tumor mutational burdens (TMB) in the range of 1 to 14 mutations/mb and a median TMB of 4.65 mutations/mb (Table 3).

In this study, 55.81% (*n* = 24) of patients found to harbor ALK alterations were initially diagnosed with comprehensive NGS. Of the remaining patients, nine (20.93%) had ALK alterations identified via FISH, six patients (13.95%) via RT-PCR, and one patient (2.33%) via IHC. There were three (6.98%) additional patients noted to have ALK alterations detected at an outside facility prior to referral to our institution. However, pathology records were unavailable and could not be obtained, and an ALK alteration was not detected on repeat molecular testing at our facility (Table 4).

**Table 4:** Initial ALK alteration identification by molecular testing

Total ALK alterations (*n* = 43)	IHC (%)	FISH (%)	RT-PCR (%)	NGS (%)	Detected at an outside facility but not confirmed on our testing (%)
ALK alterations	1 (2.33)	9 (20.93)	6 (13.95)	24 (55.81)	3 (6.98)

Multivariate logistic regression showed that Hispanic ethnicity [HR, 2.393; 95% confidence interval (CI), 1.115–5.092] and age at diagnosis (HR, 0.9325; 95% CI, 0.9081–0.9558) were significant variables in ALK alteration incidence (Table 5).

**Table 5:** Logistic regression for ALK alterations evaluating Hispanic ethnicity and controlling for site of treatment, age at initial diagnosis, gender, and smoking

Dependent variable		OR	95% CI
ALK alteration	Smoking: yes (vs. no)	0.5511	0.2561–1.161
	**Ethnicity: Hispanic (vs. non-Hispanic)**	**2.393**	**1.115–5.092**
	Gender: male (vs. female)	1.572	0.7643–3.275
	Age at initial diagnosis	0.9325	0.9081–0.9558
	Site of treatment: Norris (vs. LAG)	0.7931	0.3607–1.682

**Figure 1:**
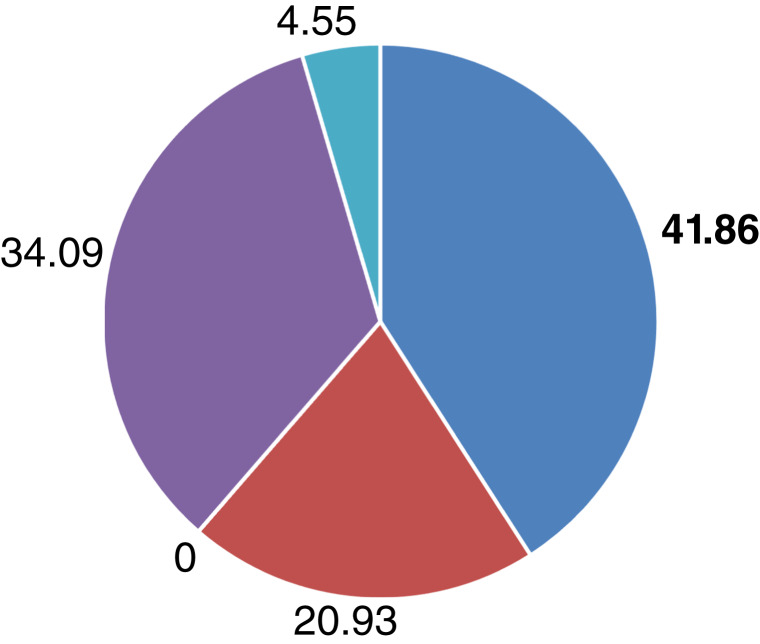
Distribution of ALK alterations by race/ethnicity. Percentage of all ALK alterations (*n* = 43) by race/ethnicity. Blue, Hispanic; red, Asian; purple, non-Hispanic White; and turquoise, other/unknown. No Non-Hispanic Blacks had ALK alterations.

Of the 43 patients who were found to have ALK alterations, 18 (41.86%) of the patients were Hispanic compared with 14 (32.56%) non-Hispanic White, nine (20.93%) Asian, and two (4.55%) other/unknown (Fig. 1).

**Figure 2:**
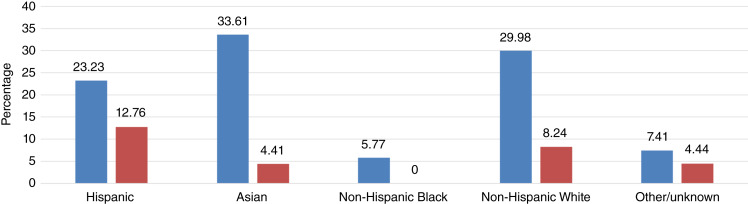
Race/ethnicity distribution in 607 patients with NSCLC and 43 ALK alterations by percentage. Blue, percentage of specific race/ethnicity in the total population of NSCLC; red, percentage of ALK alterations by race/ethnicity in the total population of NSCLC.

Despite Hispanics only accounting for 23.23% of the NSCLC population in this study, there was an ALK prevalence of 12.76% compared with the Asians comprising 33.61% of the NSCLC population and having an ALK prevalence of 4.41%, non-Hispanic Blacks comprising 5.77% of the NSCLC population and having an ALK prevalence of 0%, non-Hispanic Whites comprising 29.98% of the NSCLC population and having an ALK prevalence of 7.69%, and other/unknown subgroups comprising 7.41% of the NSCLC population and having an ALK prevalence of 4.44% (Fig. 2).

## Discussion

There is limited information about disparities in genomic mutational analyses among various racial groups. Previous studies have highlighted that Hispanics/Latinos exhibit a higher prevalence of actionable mutations compared with non-Hispanic Whites, aligning more closely with mutational frequencies observed in Asian populations (24). This study specifically investigates the incidence of ALK alterations across different racial groups at a large academic center in Los Angeles, California, aiming to elucidate the underlying factors shaping their mutational profiles. At the time of this review’s publication, this is the first study evaluating the incidence of ALK alterations across racial groups, including Hispanic/Latino patients specifically in California.

Since the first description of ALK gene rearrangements in NSCLC by Soda in 2007, there has been a wide range of ALK incidence noted in the literature (<2%–13%; refs. 25, 26), and the incidence of ALK alterations of 7.08% (*n* = 43/607) observed in this study fits within the previously observed range. Similar to prior data, this study also observed that patients who harbored ALK alterations were found to have a lower median age at the time of diagnosis when compared with the total population (53.0 vs. 65.7 years). In addition, 62.79% (*n* = 27/43) of patients harboring ALK alterations were never smokers and 86.05% (*n* = 37/43) were diagnosed with lung adenocarcinoma (Table 3).

Upon further stratification by race/ethnicity, Hispanic patients were more likely to present with stage IV disease at diagnosis when compared against the total population (73.05% vs. 57.66%). According to the California Cancer Registry, similar trends have been reported across California. Of patients diagnosed with lung cancer in California between 2012 and 2021, 58.8% and 51.7% of Hispanic men and women presented with metastatic disease at the time of diagnosis compared with 50.3% and 46.3% of non-Hispanic White men and women, respectively (CAL*Explorer, RRID: SCR_026228). Similarly, in a recent study of 1.5 million patients with NSCLC, all Hispanic groups were more likely to present with later-stage cancer; however, Hispanic Black patients had the highest proportion of metastatic NSCLC at diagnosis followed by Hispanic White and Hispanic others, highlighting the shortcomings of treating these groups as a monolith (27). The higher incidence of late-stage disease may be partially attributed to the fact that Hispanic patients tend to experience greater health disparities than non-Hispanic Whites because of structural, sociodemographic, psychosocial, and cultural factors (28). Our study also demonstrated higher rates of never smokers within the Hispanic cohort at 53.90% (*n* = 76/141) when compared against the non-Hispanic White (30.22%, *n* = 55/182) and non-Hispanic Black cohorts (22.86%, *n* = 8/35; Table 1). Similar trends have been identified in prior studies. In a prospective cohort study of 82,408 patients in California and Hawaii who self-identified as African American, Native American, Latino, Japanese American, or White, Latino men and women demonstrated some of the highest rates of never smokers at 32.5% and 66.3%, respectively. Given these public health data about higher rates of late-stage disease and never smokers within the Hispanic population and that previous literature has shown ALK^+^ patients to be predominantly never smokers who present at late stage, this correlates with our study findings showing that Hispanic patients were significantly more likely to harbor ALK alterations compared with non-Hispanic patients (5.36%, *n* = 25/466; *P* = 0.0046).

On further analyses, our study observed that non-Hispanic Whites had an ALK frequency of 7.69% (*n* = 14/182) and Asians of 4.41% (*n* = 9/204). Given the higher proportion of never smokers within our Asian cohort, one would have anticipated a higher incidence of ALK alterations. However, when factoring in logistic regression to control for smoking history, gender, and treatment site, Hispanic patients had a significantly higher prevalence of ALK mutations than non-Hispanics, suggesting that race and ethnicity are key determinants and warrant further investigation. Several studies have already explored the potential role of race and ethnicity in the prevalence of oncogenic driver mutations (29–31). However, there remains limited information on the heterogeneity of these genetic alterations among and within different racial groups and ethnicities experiencing NSCLC.

In our study, the frequency of ALK alterations noted among the Hispanic population was on the higher end of the range previously reported in the literature (<2%–13%; refs. 25, 26). Some of this variance is likely attributed to the fact that Hispanics are not a race but a diverse group of people with regional and cultural differences contributing to significant variation. The Hispanic/Latino population is comprised of a mix of Amerindian, Asian, African, and Caucasian ancestries with different proportions in different regions (32). For example, countries such as Mexico and Peru are associated with higher prevalence of patients with Amerindian and Asian ancestries when compared with countries such as Colombia and Venezuela, which see a higher prevalence of Caucasian patients (32). In a systematic review and meta-analysis of the prevalence of oncogenic driver mutations in Hispanic/Latino patients with lung cancer around the world, the overall frequency of the ALK rearrangement was estimated to be 5% (95% CI, 4%–6%; ref. 33). However, when evaluated in Latin America, the prevalence varied greatly across countries, with a prevalence of 3% in Chile (95% CI, 1%–9%), 4% in Brazil (95% CI, 3%–6%), and 11% in Peru, suggesting a broad genetic variation across regions (33). Table 6 lists several additional studies evaluating ALK prevalence across various Hispanic/Latino populations throughout Latin America and in Florida and Puerto Rico in the United States (18–21, 34–57). Of note, according to the U.S. Census Bureau (RRID: SCR_011587), more than 70% of Los Angeles County’s Hispanic population is comprised of Mexican descent. Of note, our reported incidence correlates with a study of patients in the western region of Mexico in which ALK^+^ patients were seen in 11%, which suggests that our findings tie into where patients migrate from in Latin America (57). The conglomerate of findings below highlights that the different ethnicities of Latin American countries likely correlate with the differences that we see in ALK^+^ patients both in those countries and in the United States, where the origin of the Hispanic population is very different in California compared with that in Florida. For example, a recent study of patients with NSCLC in South Florida only showed a prevalence of 1.3% ALK positivity in Hispanic patients (47).

**Table 6:** Reported ALK prevalence across the Hispanic populations in Latin America and Florida/Puerto Rico from literature search

Patient population (*n*)	Reported ALK prevalence (%)	Location of study	References
84	4	Argentina	Recondo and colleagues (34)
95	4.2	Argentina	Perez-Mesa and colleagues (20)
95	10.5	Mexico	Cruz Rico and colleagues (19)
200	5	Argentina	Pilnik and colleagues (35)
131	6.1	Argentina	Verzura and colleagues (36)
342	1.5	Argentina	Salanova and colleagues (37)
462	2.4	Argentina, Colombia, Chile, and Uruguay	Martin and colleagues (38)
933	3.64	Brazil	Gelatti and colleagues (39)
513	5.4	Brazil	Mascarenhas and colleagues (40)
350	4	Brazil	Andreis and colleagues (41)
115	7	Brazil	Montella and colleagues (42)
325	6.5	Brazil	Novaes and colleagues (43)
173	10.4	Brazil	De Oliveira and colleagues (44)
125	4.8	Brazil	De Melo and colleagues (45)
62	3.2	Brazil	Lopes and colleagues (46)
4,697	6.8	Argentina, Chile, Colombia, Costa Rica, Mexico, Panama, Peru, Uruguay, and Venezuela	Arrieta and colleagues (18)
1,643	4.5	Chile, Brazil, and Peru	Rivas and colleagues (48)
52	5.8	Chile	Fernandez-Bussy and colleagues (49)
392	4.6	Colombia	Alarcon and colleagues (50)
79	3.8	Ecuador	Fernandez Freire and colleagues (51)
656	3.5	Genie Consortium	Velazquez and colleagues (52)
450	3.77	Mexico	Lopez Resendiz and colleagues (53)
85	6.7	Mexico	Hernandez-Pedro and colleagues (54)
710	3.9	Puerto Rico	Zheng and colleagues (21)
492	5	Florida	Raez and colleagues (55)
748	1.3	Florida	Pinheiro and colleagues (47)
29	24.13	Venezuela	Sanchez Anaya and colleagues (56)
57	11.1	Mexico	De Jesus Mora Pineda and colleagues (57)

This heterogeneity in incidence and prevalence is further complicated by the place of nativity and residence, given the interplay between genetics and environment, including occupational exposures, radiation, pathogens, and lifestyle factors (diet, obesity, and smoking). Previous studies comparing cancer outcomes between similar migrant populations across different geographic locations have underscored this gene–environment interaction. In a review of mortality data from 2008 to 2012 in Mexico and California, Mexican American men and women had 49% and 13% higher mortality than their counterparts in Mexico, respectively (58). However, for Mexican immigrants, overall cancer mortality was similar to that in Mexico (58). For lung cancers, the annual age-adjusted mortality rates per 100,000 were 15.4 (95% CI, 15.2–15.7) for Mexicans in Mexico, 23.9 (95% CI, 22.7–25.3) for Mexican immigrants in the United States, and 30.1 (95% CI, 28.5–31.8) for Mexican Americans (58). Unfortunately, our study was limited in our ability to identify and analyze distinct outcomes between different Hispanic subgroups because of incomplete data about the country of birth and history of residence.

Another factor that may contribute to differences observed between and among various racial and ethnic groups is differences in the tumor microenvironment. In a recently published large-scale transcriptomic profiling of tumor microenvironments in 5,490 patients with NSCLC, ALK^+^ tumors were associated with high PD-L1 (>50%) expression (40% vs. 47% for *KRAS*-mutated vs. 18% for *EGFR*-mutated tumors; *P* < 0.001) when compared with *EGFR*- and *KRAS*-mutated tumors. However, despite higher PD-L1 expression, ALK^+^ tumors also harbored several features of inert immune tumor microenvironments, including decreased CD8^+^ T cells (fold change −1.3; *P* < 0.001) and lower TMB (median 3 mutations/mb) when compared with *KRAS*^+^ and *EGFR*^+^ tumors (nine for *KRAS*-mutated vs. four for *EGFR*-mutated tumors; *P* < 0.001; ref. 59). Beyond these differences between gene-arranged subtypes of NSCLC, the literature has shown significant racial disparities within tumor microenvironments. In a recent study of the tumor microenvironment in patients with residual breast cancer, Black patients were found to have higher tumor microenvironment of metastasis doorway densities, which were associated with poorer prognosis (60). Tumor microenvironment of metastasis doorways are cellular structures composed of actin regulatory protein mammalian-enabled–expressing tumor cells, perivascular macrophages (TIE2-high), and endothelial cells that serve as portals for surrounding tumor cells to intravasate into bloodstream and affect other organs (60). In another analysis of 280 patients with NSCLC, African American patients were found to have a higher proportion of plasma cell signatures within the tumor microenvironment when compared with non-Hispanic Whites (61). Furthermore, gene expression patterns in African American PD-L1–positive tumors suggested higher numbers of γδ T cells and resting dendritic cells and lower numbers of CD8^+^ T cells after adjusting for age, sex, pack-years, stage, and histology (61). Further investigation of racial variation within tumor microenvironments, especially in Hispanic patients with NSCLC, is required at this time.

There are several limitations that have impacted this study. The sample size of patients with ALK alterations at our institution is relatively small, potentially limiting the generalizability of these findings. As such, our study included only a small number of distinct EML4–ALK variants. A greater frequency of variant 1 (66.67%) was observed when compared with variants 2 (6.66%) and 3 (26.67%). This contrasts with the findings of Lara-Mejia, which reported variant 3 as the most identified subtype in Hispanic patients (12). Another limitation is the reliance on self-reported ethnicity reported in chart reviews as this introduces the possibility of subjective reporting bias. Furthermore, understanding the nativity of patients and history of residence would provide additional valuable insights into the distribution of targetable mutations; however, this information was not obtained in this study.

Lastly, due to the restricted availability of CGP in a resource-limited setting, patients received various forms of mutational testing to identify ALK alterations (comprehensive profiling vs. a la carte testing for individual alterations), which may have contributed to differences in the incidence of targetable mutations. Only 55.81% (*n* = 24) of this cohort had ALK alterations detected via comprehensive NGS. Of the remaining cohort, nine patients (20.93%) had ALK alterations identified by FISH, six patients (13.95%) by RT-PCR, and one patient (2.33%) by IHC (Table 4). In a recent comparison of the EML4–ALK fusion gene positivity rate in different detection methods in samples of NSCLC, RT-PCR had the highest positivity rate at 11.62%, followed by IHC at 9.51%, and NGS had the lowest positivity rate at 5.85% (62). Similar results were observed in a cohort of 200 patients with NSCLC, in which RT-PCR yielded the highest EML4–ALK positivity at 12.5% versus IHC and FISH analysis, which had positivity rates of 6.7% and 4.5%, respectively (63). In conclusion, these findings highlight the variability in detection methods for ALK alterations and underscore the importance of utilizing the most effective testing strategies to ensure accurate identification of targetable mutations in resource-limited settings.

### Conclusion

This study reveals a significantly higher incidence of ALK alterations among Hispanic patients with NSCLC at a large academic center in Los Angeles, California, with a prevalence of 12.76% compared with 5.36% in non-Hispanic patients. This finding underscores the influence of race and ethnicity on the incidence of molecular alterations in NSCLC. The higher frequency of ALK alterations among Hispanics compared with that in other racial groups in our cohort aligns with previous studies and highlights the need to increase access to genomic profiling in this population. Further analysis of Hispanic subgroups in the literature shows that there is considerable variability in ALK alteration prevalence, potentially influenced by environmental factors and biological factors such as nativity and tumor microenvironment, illustrating the multifaceted nature of molecular alterations.

Despite the study’s limitations, including the small sample size and variations in mutational testing methods, the data provide important insights into the disparities seen in ALK alterations between different racial groups. Future research should aim to investigate the prevalence of ALK alterations in a larger, more diverse patient cohort. It would also be important to collect information on the country of birth and history of residence of patients to assess the potential influence of environmental factors on the presence of actionable mutations. Additionally, exploring the role of tumor microenvironments in mutation prevalence will enhance our understanding of the biological factors influencing these alterations. These efforts will be crucial for understanding the full scope of factors affecting the prevalence of ALK alterations, which will lead to addressing the disparities seen in various racial/ethnic groups and improving treatment outcomes for those with ALK^+^ NSCLC.
